# Changes in NMDA Receptor Function in Rapid Ischemic Tolerance: A Potential Role for Tri-Heteromeric NMDA Receptors

**DOI:** 10.3390/biom12091214

**Published:** 2022-09-01

**Authors:** Mian Xie, Tiandong Leng, Samaneh Maysami, Andrea Pearson, Roger Simon, Zhi-Gang Xiong, Robert Meller

**Affiliations:** 1 Neuroscience Institute, Morehouse School of Medicine, 720 Westview Dr SW, Atlanta, GA 30310, USA; 2 Department of Neuroscience, School of Life Sciences, Keele University, Staffordshire, Keele ST5 5BG, UK

**Keywords:** NMDA receptor, ischemic preconditioning, ischemic tolerance, oxygen and glucose deprivation

## Abstract

In this study, we characterize biophysical changes in NMDA receptor function in response to brief non-injurious ischemic stress (ischemic preconditioning). Electrophysiological studies show NMDA receptor function is reduced following preconditioning in cultured rat cortical neurons. This functional change is not due to changes in the reversal potential of the receptor, but an increase in desensitization. We performed concentration–response analysis of NMDA-evoked currents, and demonstrate that preconditioned neurons show a reduced potency of NMDA to evoke currents, an increase in Mg^2+^ sensitivity, but no change in glycine sensitivity. Antagonists studies show a reduced inhibition of GluN2B antagonists that have an allosteric mode of action (ifenprodil and R-25-6981), but competitive antagonists at the GluR2A and 2B receptor (NVP-AMM077 and conantokin-G) appear to have similar potency to block currents. Biochemical studies show a reduction in membrane surface GluN2B subunits, and an increased co-immunoprecipitation of GluN2A with GluN2B subunits, suggestive of tri-heteromeric receptor formation. Finally, we show that blocking actin remodeling with jasplakinolide, a mechanism of rapid ischemic tolerance, prevents NMDA receptor functional changes and co-immunoprecipitation of GluN2A and 2B subunits. Together, this study shows that alterations in NMDA receptor function following preconditioning ischemia are associated with neuroprotection in rapid ischemic tolerance.

## 1. Introduction

N-methyl-D-aspartate (NMDA) receptors have a dichotomous nature, being both pivotal to synaptic plasticity [1] and a significant mediator of neurotoxicity following acute brain injury, such as strokes [2,3]. NMDAR antagonists are shown to be neuroprotective in multiple ischemia studies in vivo [4,5] and in vitro [6,7], however, their unfavorable toxicity profiles [8] render their use for clinical therapy challenging. Disruption of the physiological functions of NMDA receptors by such NMDA antagonists may contribute to the side effects that outweigh their neuroprotective benefits [9,10]. Therefore, searching for alternative mechanisms of reducing neuropathological NMDAR-mediated neurotoxicity, without disrupting physiological NMDA function, is of particular clinical interest.

The NMDA receptor is a tetramer comprised of two GluN1 subunits (glycine binding) and two NR2 subunits (glutamate binding). The pharmacological sensitivity and biophysical properties of Mg^2+^ sensitivity and the decay half-life of NMDA receptors are GluN2-subunit-dependent [11]. Four genes encoding NR2 subunits have been reported. Many studies of NMDA receptor function focus on the role of GluN2A and GluN2B di-tetrameric receptors as synaptic/physiological, or extrasynaptic/pathophysiological, respectively [12,13,14,15]. However, this model may be over simplistic, given that mature adult synapses contain functional GluN2B subunits, and recent evidence suggests adult synapses contain tri-heteromeric NMDA receptors (consisting of two NR1/GluN2A/GluN2B) [16]. Electrophysiological studies give further support to the significance of tri-heteromeric NMDA receptors [17,18], yet little is known about their role in pathophysiological events.

We recently reported a change in NMDA receptor function following preconditioning ischemia (a brief ischemic stress that results in neuroprotection 1 h later). NMDA receptor currents and NMDA-mediated neurotoxicity are reduced following preconditioning, due to ubiquitin–proteasome-mediated cytoskeleton remodeling [19,20]. Here, we perform a more thorough biophysiological characterization of NMDA receptor function in ischemic tolerant neurons, and suggest a potential role for tri-heteromeric NMDA receptors in the neuronal response to ischemic preconditioning.

## 2. Materials and Methods

### 2.1. Cell Culture

Cultures of primary cortical neurons were prepared according to the previously described techniques from 1 day old Sprague Dawley rat pups (either sex) [19]. All experiments were performed in accordance with American animal protection legislation and approved by the Morehouse School of Medicine Institutional Animal Care and Use Committee. Neurons were grown for 10–14 d in culture before experiments, when cultures consisted of 80–90% neurons based on NeuN staining results. For ischemia modeling, we performed oxygen and glucose deprivation (OGD). Cells were washed twice in PBS (0.5 mM CaCl_2_, 1 mM MgCl_2_; pH 7.4) and then incubated for 30 min in an anaerobic chamber (Forma Scientific, Marietta, OH) (85% N_2_, 5% H_2_, 10% CO_2_) in PBS at 37 °C. After ischemia, cells were replenished with Neurobasal A media and placed into a normoxic incubator to recover for 1 h.

### 2.2. Electrophysiology

Patch clamp recordings were performed as described previously [19]. All recordings were performed on neurons 10–14 d in culture, and OGD-treated cells were recorded 1h following recovery. NMDA, ifenprodil, and RO 25-6981 were from Tocris Bioscience (Ellisville, MO, USA). NVP-AAM077 was a kind gift from Novartis. Other reagents were from Sigma-Aldrich (St. Louis, MO, USA). For whole-cell recordings, the intracellular solution contained 140 mM CsF, 2.0 mM MgCl_2_, 1.0 mM CaCl_2_, 10 mM HEPES, 11 mM EGTA, 4 mM Mg-ATP, and 2 mM tetraethylammonium chloride (TEA-Cl) (pH 7.25–7.35, 300–310 mOsm). The extracellular solution contained 150 mM NaCl, 5.4 mM KCl, 2.0 mM CaCl_2_, 10 mM HEPES, and 10 mM glucose (pH 7.3–7.4, 325–335 mOsM). Unless otherwise specified, 1 µM tetrodotoxin and 3 µM glycine were added into all extracellular solutions. Patch electrodes were constructed from thin-walled borosilicate glass (1.5 mm diameter, World Precision Instruments, Sarasota, FL, USA) and the resistances of the electrodes were 2–4 MΩ when filled with intracellular solution. Cells were held at −60 mV, and NMDA currents were elicited by the rapid perfusion of extracellular solutions containing 100 µM NMDA, except in the NMDA concentration–response study, where the concentration of NMDA was specified per the experiment design (1–1000 µM). A multi-barrel perfusion system (ALA Scientific Instruments, Farmingdale, NY, USA) was used to achieve a rapid exchange of extracellular solutions. Whole-cell currents were recorded using Axopatch 200B amplifiers (Axon Instruments, Baltimore, MD, USA), and data were filtered at 2 kHz and digitized at 5 Hz using a Digidata 1320 DAC unit (Axon Instruments). On-line acquisition was performed using pCLAMP software (version 10, Axon Instruments). For each cell, a voltage step of −5 mV from the holding potential was applied to monitor the cell capacitance and access resistance. Recordings with an access resistance larger than 15 MΩ were excluded from data analysis.

### 2.3. Biotinylation Assay and Streptavidin-Pulldown

Biotinylation assays were performed using the EZ-Link Micro Sulfo-NHS-SS-Biotinylation Kit per manufacturer’s directions (Thermo Scientific, Bohemia, NY, USA). Briefly, for NMDA receptor internalization, cells were incubated with EZ-Link Micro Sulfo-NHS-SS-Biotin for 15 min. The cells then were washed with PBS, subjected to either 30 min OGD or sham OGD, and recovered for 1 h. Cells were washed twice with 100 mM ice-cold 2-mercaptoethanesulfonic acid (MESNA) on ice before harvesting. For external NMDA receptor labeling, cells were subjected to either 30 min OGD or sham OGD before they were labeled with EZ-Link Sulfo-NHS-LC-Biotin for 15 min in ice. The cells were then washed twice with PBS on ice and harvested. Cells were lysed and incubated with streptavidin agarose resin (Thermo Scientific) for 1 h at room temp. Lysates were washed 3 times with PBS NP40 (0.1%) and processed for immunoblotting.

### 2.4. Immunoprecipitation

Immunoprecipitation (IP) assays were performed on neurons at 12 d in culture. MagnaBind protein A magnetic beads (Thermo Scientific) were washed with PBS containing 0.1% Tween 20 using Magna GrIP Rack (Thermo Scientific) before prebinding to capture antibodies (5 µg) for 30 min at room temp. The beads were washed again 3 times before incubating with cell lysates (500 µg) overnight at 4 °C. Precipitated proteins were washed, eluted by boiling in gel-loading buffer, then subject to PAGE for immunoblotting.

### 2.5. Immunoblotting

Immunoblotting was performed as previously described [19]. Protein samples (50 μg) were denatured, subjected to PAGE, and transferred to polyvinylidene difluoride membranes (Bio-Rad, Hercules, CA, USA). Membranes were incubated with primary antibodies to GluN1 (BD Biosciences, Cayey, Puerto Rico), GluN2A (Sigma), or GluN2B (BD Biosciences) at 4 °C overnight, and then anti-rabbit or anti-mouse IgG conjugated to horseradish peroxidase (Cell Signaling Technology) for 2 h at room temperature. Chemiluminescence (Visualizer Millipore, Charlottesville, VA, USA) was captured and quantified using a Kodak Imagestation 4000 MM. 

### 2.6. Data Analysis

Data are reported as mean ± SEM. Statistical analyses were performed using one way ANOVA or two-way ANOVA, followed by Bonferroni’s multiple comparison test using GraphPad Prism version 6.0 (GraphPad Software, San Diego, CA, USA). All electrophysiology data were recorded on 3–5 independent cultures. To calculate NMDA and glycine potency, the following equation was used for curve fitting to calculate the EC_50_: *I = I_max_/[I + (EC_50_/[concentration])^nH^]* where *nH* is the empirical *Hill coefficient* and *[concentration]* represents the concentration of agonist in ECF. IC_50_ of Mg^2+^ inhibition was calculated by fitting the Mg^2+^ concentration–response curves with the equation *I = I_max_ [1 − 1/(1 + IC_50_)^nH^]*, where *I_max_* is the maxim current recorded with 3 µM glycine and *nH* is the empirical Hill coefficient. Curve fitting was performed in Sigma plot. Comparisons on NMDA current density, shift of NMDA reverse potential, and Ca^2+^ permeability ratio were performed with paired Student’s *t*-test. pEC_50_ and pIC_50_ values for NMDA potency, glycine potency, and Mg^2+^ blockage were analyzed with unpaired Student’s *t*-test (two-tailed).

## 3. Results

### 3.1. Biophysical Analysis Reveals Decreased Potency of NMDA following Preconditioning

**Figure 1:**
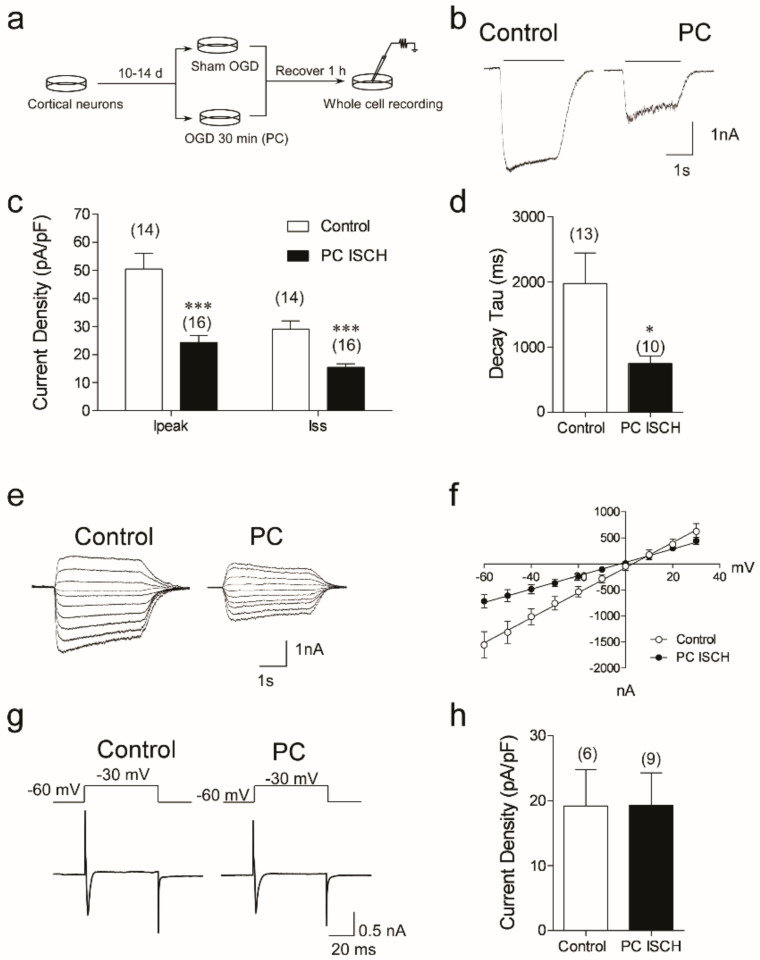
Ischemia preconditioning induces NMDA current reduction in cultured cortical neurons. (**a**) Primary cultured cortical neurons of 10–14 DIV were treated with either 30 min OGD or sham OGD followed by 1 h of recovery in normoxic incubator before they were used for whole-cell recording. (**b**) Representative traces of whole-cell currents evoked with 100 µM NMDA in control and PC neurons. Neurons were held at −60 mV and currents were recorded in ECF containing 1 µM TTX and 3 µM Gly. (**c**) The current densities of NMDA-evoked currents in control neurons calculated from peak currents (Ipeak) and steady-state current (Iss). Data shown are mean ± sem, *** denotes *p* < 0.001, Student’s 2-tail *t*-test. (**d**) Desensitization time constants in presence of 100 µM NMDA were analyzed by exponentially fitting the bottom portion of the currents shown in B. Data shown are mean ± sem, *n* = 13 in control and 10 in PC neurons (* *p* < 0.05 Student’s 2-tail *t*-test). (**e**,**f**) The I–V relationship of NMDA-evoked currents with exemplary traces in control and PC neurons. In both groups, a linear I–V relationship (calculated from Iss) is found, and the reverse potentials in both groups are around 0 mV (2.9 and −2.34 Mv). Data shown are mean ± sem; *n* = 10. (**g**,**h**) In a subset of neurons, TTX was omitted from ECF and a 10 mV voltage step protocol from −60 to +30 mV was applied to elicit voltage-evoked Na^+^ currents. The exemplary traces shown are elicited at −30 mV. Data shown are mean ± sem, *n* = 6 and 9. No statistical difference is found (*p* = 0.99, 2-tail Student’s *t*-test).

Our previous studies show NMDA-mediated peak currents are reduced in neuronal cells one hour after a preconditioning ischemic stress (30 min OGD) [19]. We performed whole-cell patch recordings in response to 100 µM NMDA from 14 DIV neurons subjected to sham OGD or 30 min OGD and recovered for one hour (Figure 1a). Both peak currents (I_peak_) and steady state currents (I_ss_) are significantly reduced in preconditioned neurons, by approximately 60% (Figure 1b,c). The NMDA-evoked current density (current normalized to cell capacitance) is also reduced in preconditioned neurons. Compared to control, following preconditioning OGD, the current density of NMDA-evoked I_peak_ is reduced by 60% (50.5 ± 5.8 pA/pF vs. 23.3 ± 2.4 pA/pF, *n* = 16, 14, *p* = 0.0001) (Figure 1c). Likewise, the current density of the steady-state current (I_ss_) is also reduced following preconditioning ischemia (29.1 ± 3.0 pA/pF vs. 15.5 ± 1.31 pA/pF, *n* = 16,14, *p* = 0.0001).

We further investigated biophysical parameters with respect to the NMDA response. The current–voltage (I–V) relationship of the NMDA receptors in control and PC-treated neurons was assessed by plotting the 100 µM NMDA-evoked I_ss_ as a function of membrane potentials in the absence of Mg^2+^ (Figure 1e,f). In both control and preconditioned cells, the I–V responses are linear and reverse near 0 mV. The major difference between the response is the lower slope in the preconditioned cells (Figure 1f). We analyzed the desensitization time constant of the 100 µM NMDA-evoked currents, by fitting the current response to a monoexponential equation (see Methods). Analysis of our data shows a decrease in decay constant tau from 1979 ± 485 ms in control cells to 748 ± 122 ms in cells subjected to preconditioning ischemia (*p* = 0.0345) (Figure 1d). These data suggest that the desensitization of NMDA-elicited currents in preconditioned cells is faster compared to control cells.

As a control, we examined the general excitability of the neurons following preconditioning ischemia, by measuring voltage-dependent Na^+^ currents using recording media containing no tetrodotoxin (TTX), and stepping the membrane potential from −60 mV to +30 mV (10 mV steps). The largest peak Na^+^ currents are recorded at −30 mV in both groups of neurons (Figure 1g). There is no difference in the current density between control and PC-treated cells (19.2 ± 6.1 pA/pF (*n* = 6) and 19.3 ± 5.3 pA/pF (*n* = 9), respectively, *p* = 0.99, 2-tail Student’s *t*-test) (Figure 1h). We found no evidence suggesting changes in the membrane integrity, such as increased leak currents (not shown). Together, these data suggest that NMDA responses in preconditioned neurons are reduced (due to smaller current amplitude and quicker desensitization to NMDA), but that the general excitability of neurons one hour following preconditioning ischemia is unchanged. 

### 3.2. NMDA Receptors Show Increased Sensitivity to Mg^2+^, but Not Glycine, following Ischemic Preconditioning

**Figure 2:**
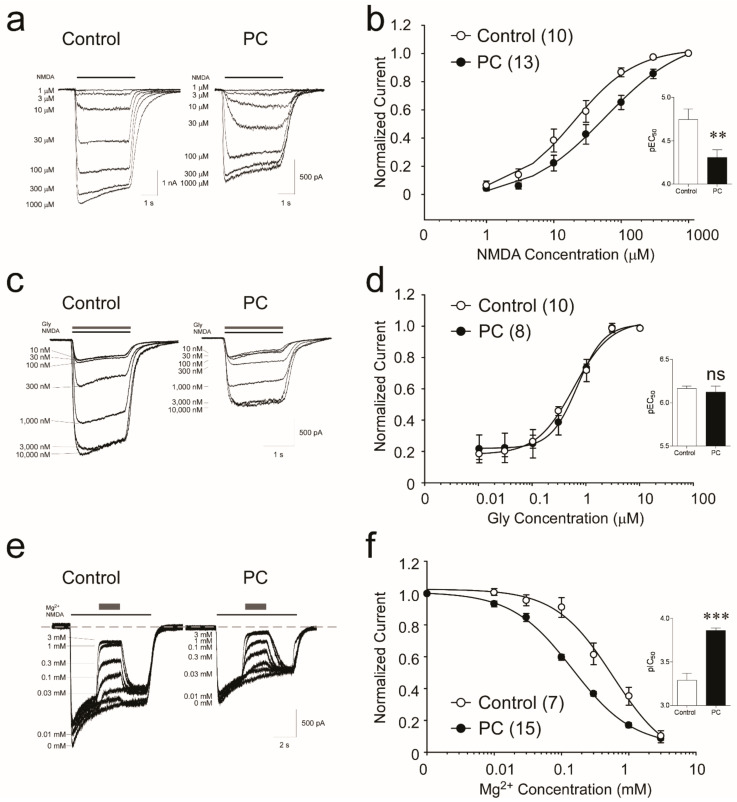
Ischemia-preconditioned neurons show decreased sensitivity to NMDA, unchanged response to Gly, and increased sensitivity to Mg^2+^ block. (**a**) Whole-cell recording was performed at −60 mV with 1 µM TTX and 3 µM Gly present. NMDA currents were evoked with 1–1000 µM NMDA (black line) in control and PC neurons. (**b**) The concentration–response relationship of NMDA-evoked currents was obtained by fitting correspondent I_ss_ with Hill equation. Inserts shown are mean ± sem of pEC_50_ values generated from individual fittings from 10 control and 13 PC neurons (** denotes *p* < 0.01, unpaired Student’s 2-tail *t*-test). (**c**) Gly sensitivity determined by applying 10–10,000 nM of Gly (grey line) with 100 µM NMDA (black line) on control and PC-treated neurons. Cells were bathed in ECF containing 1 µM TTX, but no Gly. (**d**) The concentration–response relationship of Gly-dependent currents in control and PC neurons. Insert: pEC_50_ values generated from individual fittings to Iss from 10 control and 8 PC neurons. No statistical significance is found (data are mean ± sem; *p* > 0.05, unpaired Student’s 2-tail *t*-test). (**e**) The Mg^2+^ blockage to NMDA currents was assessed by applying 0.01−3 mM of Mg^2+^ (grey bar) in the middle of NMDA application (black line) in control and PC neurons. (**f**) The concentration–response relationship was calculated by fitting the Iss values with Hill equation. Insert: pIC_50_ calculated from individual fittings of control and PC-treated neurons (*** denotes *p* < 0.001, unpaired Student’s 2-tail *t*-test).

We further examined the biophysical properties of the receptors mediating the response to NMDA. A concentration–response curve was generated to determine whether the change in response to NMDA is due to a change in potency of NMDA for the receptor (s) (Figure 2a). The calculated NMDA EC_50_ of the control neurons is 24.75 ± 6.2 µM, (r^2^ = 0.99), and in ischemia-treated neurons it increases to 62.74 ± 13.6 µM (r^2^ = 0.99) (Student’s *t-*test performed on pEC_50,_ values *p* = 0.0070, Figure 2b). These data suggest a significant decrease in NMDA potency for its receptors following preconditioning ischemia. 

NMDA receptors require the simultaneous binding of glycine to GluN1 and glutamate to GluN2 for activation [21]. In our standard experimental setting, the NMDA was co-applied with 3 µM of glycine, which is reported as a near-saturating concentration in cultured neurons [22,23]. We assessed the response of receptors to varying concentrations of glycine (0.01–10 µM) (Figure 2c). The calculated glycine EC_50_ between control and ischemia-treated neurons are not significantly different (707.7 ± 138.6 nM vs. 836.3 ± 143.9.8 nM, respectively. Student’s *t*-test on pEC_50_ values, *p* = 0.567). The Hill coefficients are also similar in control (1.87 ± 0.24) and preconditioned neurons (1.45 ± 0.16; *p* = 0.74) (Figure 2d). Hence, our results do not suggest any significant changes in glycine potency on NMDA receptors following ischemic preconditioning.

NMDA receptors are unique in their voltage-dependent blockage by extracellular Mg^2+^ [11], which is known to be directly related to the severity of excitotoxicity during ischemia [24]. To assess changes in Mg^2+^ sensitivity of receptors, we co-applied 100 µM NMDA and 3 µM glycine with 10–3000 µM of Mg^2+^ to generate Mg^2+^ concentration–response curves using a holding potential of −60 mV (Figure 2e). Compared to control neurons, we observe a left-shift of the Mg^2+^ concentration–response curve following preconditioning ischemia (Figure 2f) (Mg^2+^ IC_50_ = 581.5 ± 22.9 µM for control and 145.6 ± 13.5 µM for PC neurons, respectively, Student’s *t*-test on pIC_50_ values *p* = 0.0001) (Hill coefficients are not significantly different) (Figure 2f). These data suggest a significant increase in NMDA receptors to Mg^2+^ blockage following preconditioning ischemia.

### 3.3. Reduction in GluN2B Membrane Localization and Functional Responses in Neurons Subjected to Ischemic Preconditioning

**Figure 3:**
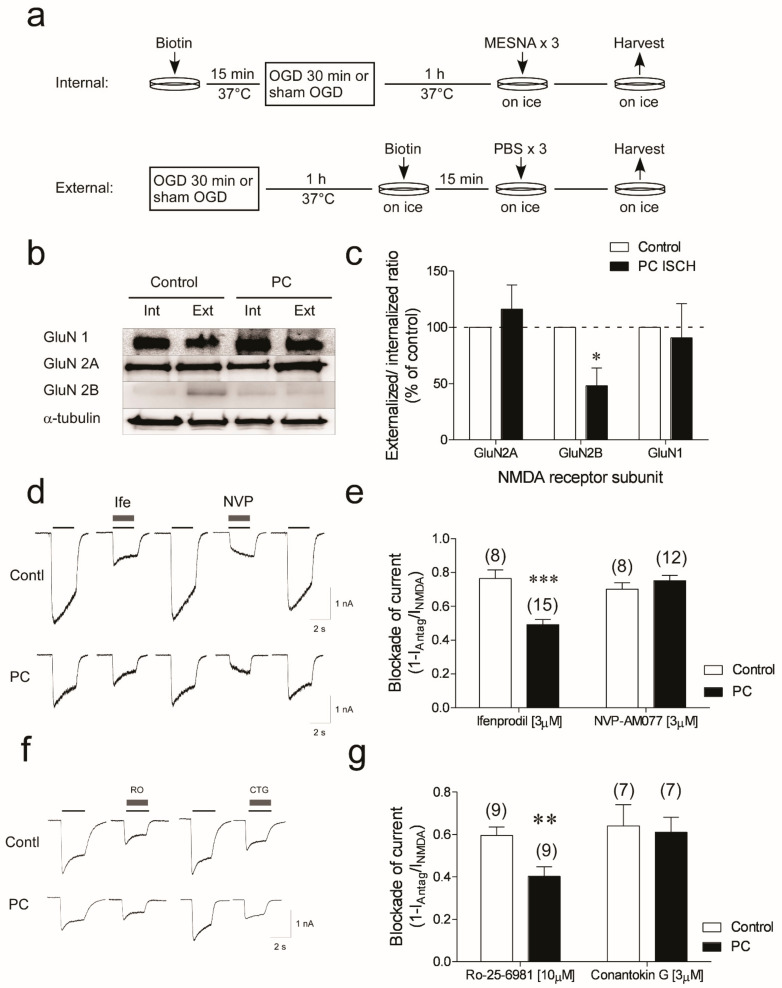
Change in membrane-located GluN2B, but not GluN1 and GluN2A subunits, and NMDA receptor antagonist efficacy following ischemic preconditioning. (**a**) Schematic drawing of surface protein biotinylation assay. The internal and external portions of cell lysate from control and PC neurons were then purified by streptavidin immunoprecipitation, and the level of target proteins were blotted with specific antibodies. (**b**) The externally and internally located GluN1, GluN2A, and GluN2B subunits in control and PC neurons shown in Western blots. (**c**) The quantified external/internal ratios of the GluN1, 2A, and 2B subunits in control and PC neurons expressed as a % of control values (data are mean ± sem; * *p* < 0.05. Student’s 2-tail *t*-test performed on raw data). (**d**) NMDA current blockage with GluN2B antagonist ifenprodil and GluN2A antagonist NVP-AAM077 in control and PC neurons. Cells were held at −60 mV, and NMDA currents were evoked with 100 µM NMDA (black line). After currents stabilized, 3 µM ifenprodil (grey bar) or 3 µM NVP-AAM077 was co-applied with NMDA. Cells were recovered by washing. In some cells, both ifenprodil and NVP-AAM077 were tested. When NVP-AAM077 was given before ifenprodil, a longer period of wash time was needed, and currents could recover to 70–90% of their initial levels. In all experiments, the solutions contained 1mM TTX and 3 µM Gly. (**e**) Inhibition profile of NVP-AAM077 and ifenprodil in control and preconditioning-ischemia-treated cells. Inhibition was determined using the formula (1-(I_antag_/I_NMDA_)). Data shown are mean ± sem (*n*) *** *p* < 0.001, 2-tail Student’s *t*-test. (**f**) Representative traces of whole-cell currents elicited by 100 µM NMDA with 3 μM of conantokin-G, which are compared with those that received 3 μM of RO25-6981 co-application in control and PC neurons. (**g**) The percent of inhibition of current (1-(I_Antag_/I_NMDA_)) calculated from I_ss_ in control and PC neurons in the presence of 3 μM conantokin-G and R0-25-6981. Data are mean ± sem (*n*) ** *p* < 0.01, 2-tail Student’s *t*-test.

The reduction in NMDA-elicited currents (Figure 1b–d) following ischemic preconditioning suggests a loss of membrane-localized NMDA receptors. The surface localization of NMDA receptors in control and ischemia-treated neurons was identified by biotinylation assay [25]. Surface proteins were cross-linked with biotin, followed by streptavidin pull-down, then blotted with specific antibodies to GluN1, GluN2A, and GluN2B (Figure 3a). We observe no significant changes in membrane-located GluN1 subunits following ischemic preconditioning (*p* > 0.05, *n* = 3). A small, but non-significant, increase in GluN2A external/internal ratio is observed following preconditioning ischemia. In contrast, following preconditioning ischemia, a significant decrease in the GluN2B external/internal ratio is evident (*p* = 0.0206, *n* = 3) (Figure 3b,c). The results indicate that one hour following ischemic preconditioning GluN2B, but not GluN2A subunits, may be removed from the cellular membrane.

The potential role of GluN2B was assessed using ifenprodil, a non-competitive GluN2B antagonist [26]. NMDA (100 µM) currents are reduced by 76.5% in control cells, but only 49.2% in preconditioned neurons (Figure 3d,e) (Control: 42.5 ± 6.1 pA/pF to 10.8 ± 3.8 pA/pF (76.5% ± 5% inhibition); PC-treated: 22.0 ± 3.1 pA/pF to 11.7 ± 1.8 pA/pF, a 49.2 ± 2.9% inhibition); *p* = 0.0001). This suggests a loss in potency of ifenprodil to block GluN2B-mediated responses in preconditioned neurons. We observe a similar loss in inhibition of NMDA-evoked currents in preconditioned neurons with a second non-competitive GluN2B antagonist, RO 25-6981 (Control: 59.6% ± 4% vs. PC-treated: 40.3 ± 4.5%; *p* = 0.0054) (Figure 3f,g). We assessed a competitive, GluN2B-selective antagonist, conantokin-G [27,28] (that also inhibits GluN1/GluN2A/GluN2B tri-heteromeric NMDA receptors [29,30]). Conantokin-G (3 µM) is equally effective at blocking NMDA-evoked currents in controls and PC-treated neurons (61.1% ± 7% inhibition vs. 64% ± 9.9% inhibition, respectively; *p* = 0.80) (Figure 3f,g). Therefore, following preconditioning ischemic stress, there is no change in the blockade of NMDA-evoked currents by conantokin-G. There is no significant change in the ability of a relatively selective GluN2A antagonist NVP-AAM0077 [31] (3 µM) to block NMDA-evoked currents in preconditioned neurons (Control: 33.2 ± 2.1 pA/pF to 10.9 ± 1.8 pA/pF (70.2% ± 4% inhibition); PC-treated 21.6 ± 1.6 pA/pF to 8.0 ± 1.8 pA/pF (75.2% ± 3% inhibition); *p* = 0.31) (Figure 3d,e). Together, these data suggest preconditioned neurons are less sensitive to non-competitive (allosteric) NMDA receptor antagonists, but not competitive antagonists.

### 3.4. Evidence for Increased Formation of Tri-Heteromeric NMDA Receptors following Ischemic Preconditioning

**Figure 4:**
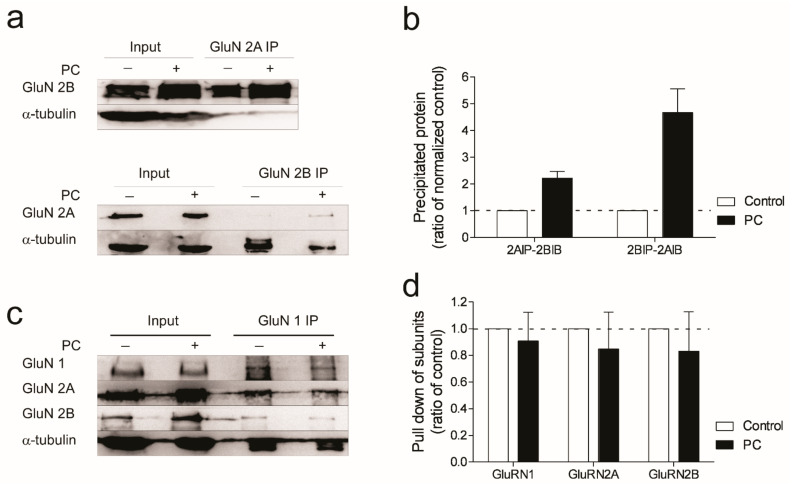
Increase of tri-heteromeric NMDA receptors following ischemic preconditioning. Cultured neurons of 12–13 DIV were treated with either sham OGD or 30 min OGD followed by 1 h recovery in the normoxic incubator before they were used for immunoprecipitation assay. (**a**) Immunoblots of cell lysates subjected to immunoprecipitation with GluN2A or GluN2B antibody. (**b**) Quantification of data from 3 IP assays. Data were normalized to input and expressed as a ratio of control IPs (mean ± sem). (**c**) Immunoblots of cells lysates subjected to immunoprecipitation with GluN1 antibody and blotted with antibodies specific to GluN2A and 2B. Similar levels of GluN2A and 2B are found in the control and PC neurons. (**d**) Quantified data from 3 independent assays show that no significant change is found between the control and PC neurons in regard to either GluN2A or GluN2B.

Recent studies show functional tri-heteromeric conformations of NMDA receptors (NR1/GluN2A/GluN2B) to be present in mature brains [16,18]. We investigated the assembly of NMDA receptors using GluN2A- and GluN2B-subunit-specific immunoprecipitation. We show an increase in the immunoprecipitation of GluN2B when incubating lysates with a GluN2A bait antibody following preconditioning ischemia (Figure 4a,b). We confirm this observation by reversing the IP and using GluN2B antibodies as the bait in the IP reaction (Figure 4a,b). Since we do not detect any significant decrease in the levels of GluN1–GluN2A or GluN1–GluN2B-complexed proteins (Figure 4c,d), we conclude the increase is not likely due to a separation of the GluN2A/GluN2B complex from the obligatory GluN1 subunits.

### 3.5. Stabilizing Actin Polymerization Partially Lessened the NMDA Current Reduction and Blocked Formation of Tri-Heteromeric NMDAR in Preconditioned Neurons

**Figure 5:**
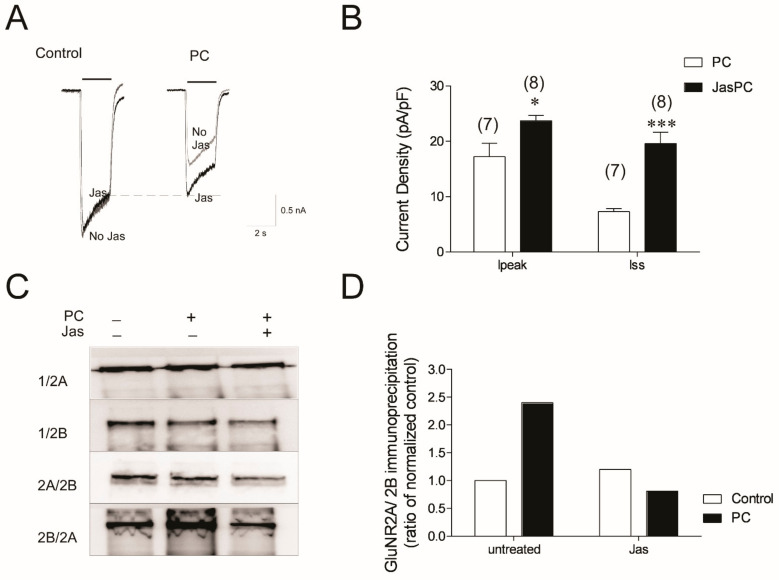
Actin stabilizer jasplakinolide partially alleviates the NMDA current reduction and reduces the formation of tri-heteromeric NMDAR in preconditioned neurons. (**A**) Cortical neurons were incubated with 3 µM of jasplakinolide for 1 h prior to the 30 min OGD or sham OGD. The whole-cell currents from the jasplakinolide-treated cells (black traces) were compared to those recorded from untreated cells from their sister cultures (grey graces). (**B**) Peak (Ipeak) and steady-state (Iss) current densities from control (white) and preconditioned cells in the presence or absence of jasplakinolide. Data shown are mean ± sem. *n* = 6 * denotes *p* < 0.05 vs. PC-only-treated cells *** denotes *p* < 0.001 vs. PC-only-treated cells. (**C**) Control, or PC neurons with and without 3 µM of jasplakinolide incubation were used in immunoprecipitation assay. Samples were immunoprecipitated with GluN1, GluN2A, or GluN2B antibody, and blotted with GluN2A and GluN2B antibody (shown as IP antibody/blot antibody). (**D**) Quantification of the blots in (**C**).

The actin-mediated stability of the synaptic structure is closely related to the activity of glutamate receptors [19,32]. Jasplakinolide prevents actin remodeling and rapid ischemic tolerance following preconditioning ischemia [19]. Jasplakinolide blocks preconditioning-induced reduction in NMDA-evoked peak and steady-state currents in neurons (*p* = 0.02 and *p* = 0.0001, respectively) (Figure 5A,B). Jasplakinolide incubation alone does not significantly affect NMDA current densities in control cells (*p* > 0.05) (Figure 5A). Jasplakinolide reduces the co-immunoprecipitation of the GluN2A–GluN2B complex in PC-treated neurons (Figure 5C,D). Together, these data suggest that blocking changes in the actin cytoskeleton following preconditioning ischemia prevents changes in NMDA receptor function, and, consequently, rapid ischemic tolerance.

## 4. Discussion

Here, we reveal a novel mechanism by which NMDA-receptor-mediated signaling is selectively attenuated following brief exposure to non-injurious ischemic stress that induces an innate protective phenotype (aka preconditioning). Following a detailed characterization of NMDA electrophysical properties in preconditioned neurons, we observe a reduction in NMDA potency, an increased desensitization rate, and an increase in the potency of Mg^2+^ to block the receptor. Inhibitor studies show a reduced inhibition of NMDA-evoked current that is blocked by non-competitive antagonists, but no change in competitive inhibition. We show a reduction in cell surface NMDA receptor GLuN2B subunits, and an increased co-immunoprecipitation of GluN2A with GluN2B subunits, which is consistent with the replacement of di-heteromeric GluN2B receptors with tri-heteromeric GluN1/GluN2A/GluN2B receptors. The reduction in signaling through NMDA receptors is mediated by dynamic changes in the actin cytoskeleton system. These transient structural changes in NMDA subunit composition result in neuroprotection to ischemia, and represent a novel approach to regulating NMDA-receptor-medicated excitotoxicity during harmful ischemia.

It is conventionally considered that the activation of GluN2B- vs. GluN2A-containing NMDA receptors have deleterious and physiological effects, respectively. However, it is not understood how the subunit complement of NMDA receptors change following preconditioning ischemia. We demonstrate that the reduction in NMDA responses following ischemic preconditioning is associated with the functional loss of NMDA receptors composing GluN2B subunits from the cell membrane, and an increased assembly of GluN1/GluN2A/GluN2B tri-heteromeric NMDA receptors. The loss in function of GluN2B-containing NMDA receptors is likely due to multiple factors. First, as shown in our biotinylation study, ischemic preconditioning results in a rapid decrease in GluN2B subunits in the neuronal cell membrane. This is probably due to increased subunit internalization and/or decreased membrane insertion following preconditioning. Consistent with this hypothesis, our previous study shows GluN2B subunits rapidly detach from the actin cytoskeleton following preconditioning ischemic stress [19]. Secondly, membrane-located NMDA receptors show a reduced potency to NMDA, and increased desensitization following ischemic preconditioning (Figure 1). As GluN2B-composing NMDA receptors are largely associated with the extrasynaptic NMDA receptors [33], and related to harmful outcomes during ischemia [13,14], a reduction in these receptors in the NMDA complex may result in a decreased excitotoxicity during ischemia, while retaining the ability of the NMDA receptor to perform physiological functions.

Conantokin-G (CGX-1007) is a snail venom peptide that competitively blocks GluN2B subunit receptors [27,28,30,34,35], but not GluN2A-containing receptors (for exception, see [36]). The effect of conantokin-G on tri-heteromeric receptors is more complex. Several studies report a partial blockade of NR1/GluN2A/GluN2B-mediated currents by conantokin-G [29,30,35], except for a recent study by Cheriyan et al. that reports conantokin-G to be of very weak potency for such tri-heteromeric receptors (5% inhibition) [37]. One potential reason for this observation is the use of a tri-heteromeric expression system, whereby the C terminus domains contain C1 and C2 tags [37,38,39]. In our study, we do not see any reduction in NMDA current inhibition in the presence of conantokin-G. Interestingly, we also see no decrease in the inhibition of NMDA-mediated currents by a second competitive NMDA receptor antagonist, NVP-AMM1077. NVP-AMM1077 was originally described as a GluN2A-selective antagonist [40], but more receptor studies suggest its selectivity is less than originally reported [31]. Although the selectivity of NVP-AMM1077 is modest when tested with glutamate as the agonist, greater selectivity is observed when NMDA is used as the agonist [31]. It should be noted that we observe similar inhibition of NMDA-mediated effects at 10, 3, and 0.1 µM in control neurons and following preconditioning ischemia (TD personal communication: not shown). Our data are consistent with previous studies showing a reduced inhibition of NMDA tri-heteromeric receptors by ifenprodil compared to di-heteromeric NMDA receptors containing GluN2B subunits [38,39]. Indeed, both non-competitive NMDA 2B receptor antagonists tested in our study show reduced inhibitory effects following ischemic preconditioning (Figure 3). A potential role for tri-heteromeric NMDA receptors is also supported by co-immunoprecipitation data (Figure 3) and the change in GluN2B–GluN2A ratios on the cell surface. The exact role of tri-heteromeric receptors in rapid ischemic tolerance will be unclear until a selective small molecule inhibitor of this receptor is identified.

The exact biophysical properties of GluN1/GluN2A/GluN2B tri-heteromeric NMDA receptors are still under investigation [17]. In our experimental setting, the NMDA EC_50_ of the control neurons is 20.0 ± 2.8 µM, which is lower than that measured from exogenously expressed recombinant rat NMDA receptors composed of GluR1/GluN2B (30 µM). In neurons receiving ischemic preconditioning, the EC_50_ increases 2.9-fold to 57.3 ± 9.6 µM, which is lower than the EC_50_ values measured from exogenously expressed recombinant rat NMDA receptors composed of GluR1/GluN2A (95 µM), but higher than the EC_50_ from GluR1/GluN2B, GluR1/GluN2C (22 µM), and GluR1/GluN2D complexes (7.3 µM) [41]. Although it is challenging to draw firm conclusions based on the biophysical properties of our recordings, due to the complexity of the neuronal system we used, the change in EC_50_ does remain in line with our hypothesis that changes in NMDA receptor subunits composition occur following ischemic preconditioning.

Different compositions of GluN2 and selective splicing of the GluN1 transcripts generate multiple receptor isoforms with distinct brain distributions and functional properties, which contribute to their complex roles in both physiological [42,43] and pathological function, including following ischemia [13,44,45]. In this study, we focused on the two major subunits of NMDA receptors: GluN2A and 2B. It is yet unknown how other NMDA receptor subunits, such as GluN3, are affected during ischemic preconditioning. The GluN3 subunits also provide glycine binding sites and co-expression of GluN3 with GluN1 and GluN2 could result in a reduction in NMDA currents [46]. The loss of GluN3A subunits containing NMDA receptors increases neuronal damage in cultured neurons following ischemia [47]. Our data do not support a role of GluN3 following ischemic preconditioning, because an unchanged glycine potency is observed following preconditioning ischemia [48]. However, we cannot rule out the possibility that the involvement of GluN3 may affect the receptor assembly, such that resultant changes in EC_50_ are very limited, due to a limited number of GluN1/GluN3 di-heteromers and/or GluN1/GluN2/GluN3 tri-heteromers being added.

The Mg^2+^ sensitivity of NMDA responses increases following ischemic preconditioning. If extracellular Mg^2+^ levels are not supra-maximal, an elevated Mg^2+^ sensitivity enhances the blockade of NMDA-receptor-mediated responses, resulting in decreased excitotoxicity. The Mg^2+^ sensitivity of NMDA receptors can be decreased by post-translational modification of the protein [49], and increased by structural changes [50]. As the binding site for Mg^2+^ is located in the pore of the NMDA receptor, how this region is affected by changes in subunits composition, such as from GluN1/GluN2A or GluN1/GluN2B di-heteromers to GluN1/GluN2A/GluN2B tri-heteromers, or by intracellular signaling events following preconditioning, is clearly worthy of further study.

Taken together, we provide evidence to support a potential role of functional GluN1/GluN2A/GluN2B tri-heteromeric receptors in the response to brief non-injurious ischemic stress. Ischemic preconditioning suppresses the excitotoxic NMDA effects [19], but CREB signaling in response to NMDA is maintained (Meller personal communication). In addition to ischemia, NMDA-receptor-mediated excitotoxicity is thought to play a critical role in many other CNS disorders, such as Alzheimer’s disease, Parkinson’s diseases, and Huntington’s disease [11]. Therefore, the preconditioning-identified alterations in NMDA receptor physiology may represent a broad mechanism, relevant to synaptic remodeling and neuronal protection, which could be applied to these neurological disorders and diseases.

## Data Availability

The datasets used and/or analyzed during the current study available from the corresponding author on reasonable request.
